# Vagus nerve stimulation enhances extinction of conditioned fear and modulates plasticity in the pathway from the ventromedial prefrontal cortex to the amygdala

**DOI:** 10.3389/fnbeh.2014.00327

**Published:** 2014-09-18

**Authors:** David Frausto Peña, Jessica E. Childs, Shawn Willett, Analicia Vital, Christa K. McIntyre, Sven Kroener

**Affiliations:** School of Behavioral and Brain Sciences, The University of Texas at DallasRichardson, TX, USA

**Keywords:** anxiety, PTSD, local field potentials, *in vivo*, LTP, LTD

## Abstract

Fearful experiences can produce long-lasting and debilitating memories. Extinction of the fear response requires consolidation of new memories that compete with fearful associations. Subjects with posttraumatic stress disorder (PTSD) show impaired extinction of conditioned fear, which is associated with decreased ventromedial prefrontal cortex (vmPFC) control over amygdala activity. Vagus nerve stimulation (VNS) enhances memory consolidation in both rats and humans, and pairing VNS with exposure to conditioned cues enhances the consolidation of extinction learning in rats. Here we investigated whether pairing VNS with extinction learning facilitates plasticity between the infralimbic (IL) medial prefrontal cortex and the basolateral complex of the amygdala (BLA). Rats were trained on an auditory fear conditioning task, which was followed by a retention test and 1 day of extinction training. Vagus nerve stimulation or sham-stimulation was administered concurrently with exposure to the fear-conditioned stimulus and retention of fear conditioning was tested again 24 h later. Vagus nerve stimulation-treated rats demonstrated a significant reduction in freezing after a single extinction training session similar to animals that received 5× the number of extinction pairings. To study plasticity in the IL-BLA pathway, we recorded evoked field potentials (EFPs) in the BLA in anesthetized animals 24 h after retention testing. Brief burst stimulation in the IL produced LTD in the BLA field response in fear-conditioned and sham-treated animals. In contrast, the same stimulation resulted in potentiation of the IL-BLA pathway in the VNS-treated group. The present findings suggest that VNS promotes plasticity in the IL-BLA pathway to facilitate extinction of conditioned fear responses (CFRs).

## Introduction

Extinction of conditioned fear is the process of attenuating fearful behavioral responses to neutral stimuli when they no longer predict aversive outcomes. Therefore, extinction requires new learning about the conditioned stimuli (CS). About 30% of individuals who experience traumatic life events develop posttraumatic stress disorder (PTSD; Nemeroff et al., 2006), which is characterized by a general impairment in the ability to extinguish fear responses (Jovanovic et al., 2010; Norrholm et al., 2011). Patients suffering from PTSD exhibit reduced ventromedial prefrontal cortex (vmPFC) activation and heightened amygdala activation (Hayes et al., 2012; Stevens et al., 2013). Similarly, experiments in rat models of fear learning suggest that the vmPFC is required for the modulation and expression of extinction memory (Sierra-Mercado et al., 2011) and that plasticity in the vmPFC-amygdala pathway underlies the suppression of fear via attenuation of central amygdala activity (Marek et al., 2013). Neuroplasticity has been observed in the basolateral complex of the amygdala (BLA) following both the formation of conditioned fear responses (CFRs; Schafe and LeDoux, 2000) and the suppression of those responses during extinction (Parkes and Westbrook, 2010; Vouimba and Maroun, 2011). Activation of the infralimbic (IL) mPFC during extinction maintains extinction plasticity in the amygdala via its projections to the BLA and intercalated cells that inhibit central nucleus activity (Pape and Paré, 2010; Amano et al., 2011; Knapska et al., 2012). Thus the encoding of fear memory and extinction results in functional changes in neurons in the BLA (Amano et al., 2011). Vagus nerve stimulation (VNS) enhances memory in rats and in humans (Clark et al., 1998, 1999). Pairing VNS with discrete stimuli or behaviors has been used to induce targeted cortical plasticity for the treatment of tinnitus (Engineer et al., 2011) and motor deficits (Porter et al., 2012), raising the possibility that VNS might also be used to direct the neural plasticity underlying extinction memory. We previously showed that pairing VNS with non-reinforced CS presentations facilitates extinction of fear responses (Peña et al., 2013). The encoding of learned events results in synaptic changes that modulate subsequent induction of plasticity, or metaplasticity. Here we examined metaplasticity in the IL-BLA pathway in animals that demonstrated VNS-enhanced extinction of conditioned fear.

## Materials and methods

### Animals and surgical procedures

**Figure 1:**
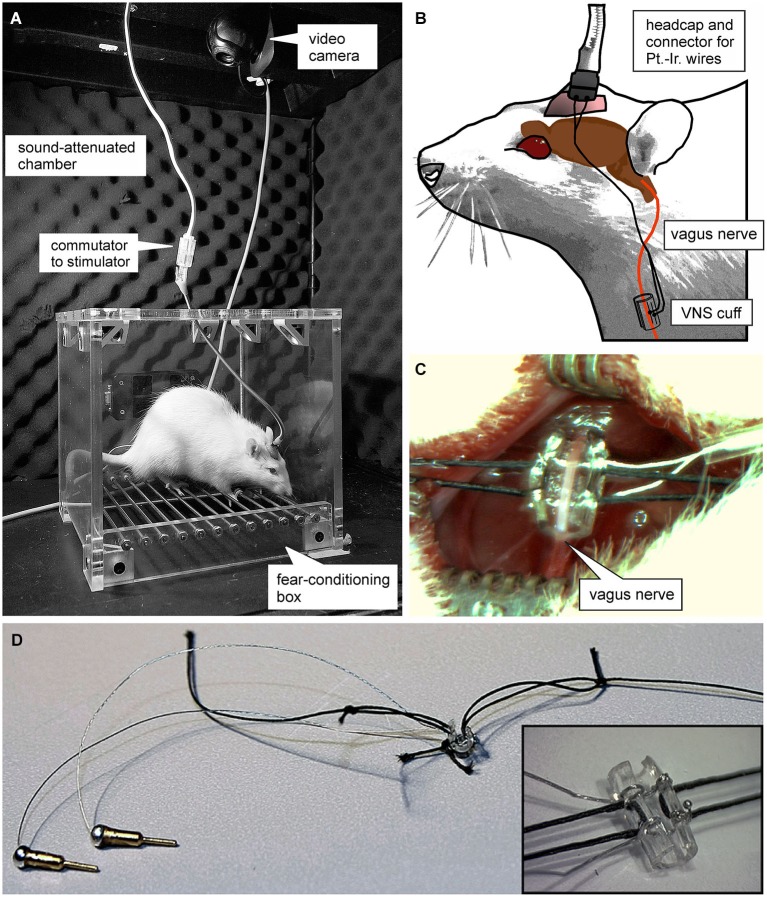
**Experimental set-up. (A)** Photograph of the plexi-glass box used for auditory fear conditioning. **(B)** Schematic of the set-up used for vagus nerve stimulation (VNS). Animals were connected to a stimulation isolation unit via a headcap from which two platinum-iridum wires lead subcutaneously to a custom-made “cuff-electrode” that is wrapped around the vagus nerve. (**C** and **D**) Photomicrographs of the cuff electrode used for VNS. **(C)** shows the surgical incision and the exposed vagus nerve before the cuff electrode is sutured around it.

All procedures were carried out in accordance with the NIH Guide for the Care and Use of Laboratory Animals, and were approved by the Institutional Animal Care and Use Committee of The University of Texas at Dallas. Male Sprague-Dawley rats (Charles River, Wilmington, MA) ~90 days old, weighing 250–300 g on arrival were housed on a 12 h light/dark cycle (lights on at 7:00 am) with access to food and water *ad libitum*. Rats were anesthetized with isoflurane (2% at an oxygen flow rate of 600–800 ml/min) and mounted in a stereotaxic frame (World Precision Instruments, Sarasota, FL). A headstage was constructed using bone screws and dental cement to fix the platinum-iridium wires used for VNS (Sigmund-Cohn Inc., Mount Vernon, NY) and a four-channel strip connector (Omnetics, Minneapolis, MN) in place. Animals were then removed from the stereotaxic and a small (1–2 cm) incision was made on the left ventral side of the neck over the jugular vein. The opening was blunt dissected until the sternomastoid, sternohyoid, and omohyoid muscles were apparent. The muscles were pulled apart using muscle retractors and the deep cervical fascia was transected to reveal the carotid sheath, containing the carotid artery and vagus nerve. The sheath was opened and connective tissue holding the vagus nerve to the carotid artery was separated for 1 cm using modified glass micropipettes. A custom-made platinum-iridium wire electrode in micro-renathane “cuff” (0.04” ID, 0.08” OD, 4 mm long) was placed around the vagus nerve (Figures 1B–D). The platinum-iridium wire leads were tunneled subcutaneously to the headstage where they connected with the inputs from an isolated pulse stimulator (MS4, Tucker Davis TechnologiesAlachua, FL). Optimal vagal fiber activation was assessed before and after completion of the study by applying VNS (0.2 mA, 60 Hz, 10 s) and observing brief cessation of breathing in anesthetized rats. Once electrode function was established, the leads were permanently fixed to their input site on the headstage with dental cement. Sham-stimulated rats were subjected to the same surgical procedure; however, cuffs were designed to not deliver current. During the 1 week recovery period, animals were handled 5 min/day for 5 days.

### Auditory fear conditioning

Auditory fear conditioning and extinction trials were performed in a plexiglas operant box (20 × 20 × 20 cm, with stainless steel grid floor, Vulintus, Plano, TX) housed in a sound-attenuated chamber (Day 1; Figure 1A). Rats were presented eight tones (9 kHz, 85 dB SPL) as CS overlapping with a 1 s footshock (0.5 mA; DSCK-C Dual Output, scrambled shocker, Kinder Scientific Co., Poway, CA), serving as the unconditioned stimulus (UCS). To prevent the development of a specific temporal association with the footshock, a single 1 s footshock was administered at a randomized time during each 30 s tone presentation. To produce robust conditioned fear, rats were again given eight tones paired with footshock on a second conditioning day 24 h later. The inter-tone-interval (ITI) was varied between 3 and 5 min, averaging 4 min for every tone presentation, in order to prevent development of a specific temporal association with the footshock.

### Conditioned fear testing

**Figure 2:**
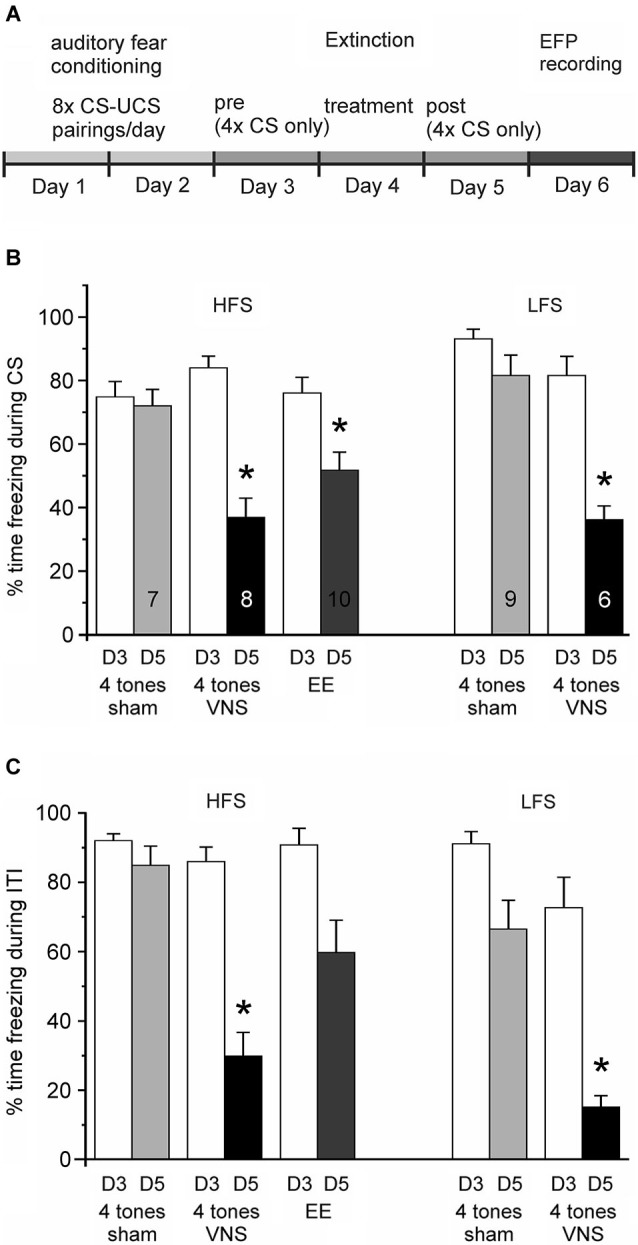
**Vagus nerve stimulation enhances extinction of auditory fear conditioning. (A)** Experimental timeline. Auditory fear conditioning consisted of 2 days of training with eight pairings between a tone (CS) and a footshock (UCS) per day. On Day 3 rats were exposed four times to the conditioned tone in the absence of footshock and freezing levels were tested (Conditioned Fear Test). On Day 4 (Treatment) rats received group-specific treatment: four tone presentations were paired with either VNS or sham-stimulation. Rats in the extended-extinction group received 20 tone presentations. Freezing levels were tested again on Day 5 in response to four presentations of the CS alone (Post Treatment test). **(B)** Percentage of time spent freezing during presentation of the conditioned stimulus (CS) on Day 3 (D3, white bars) and Day 5 (D5), respectively, for the different treatment groups (4 tones + sham stimulation, four tones + VNS, and extended extinction, EE, with 20 tones + sham stimulation). Presentation of the Day 3 and Day 5 freezing responses is separated into groups of rats in which subsequently the plasticity of the IL-BLA pathway was tested with either high-frequency (HFS) or low-frequency stimulation (LFS) of the IL. After 1 day of extinction training paired with VNS (black bar) rats spent significantly less time freezing than sham controls (^*^ *p* < 0.05). Similar levels of fear extinction were observed when rats were given extended extinction (gray bar). **(C)** Percentage of time spent freezing during the inter-tone intervals (ITI) on Day 3 (D3, white bars) and Day 5 (D5), respectively, for the same groups shown in **(B)**. Freezing levels during ITI might serve as a measure of whether extinction training generalizes to the context. Animals receiving extended extinction training did not differ significantly in their behavior from sham stimulated animals during ITIs; however, VNS animals also showed reduced freezing behavior outside the presentation of the conditioned tone.

To measure VNS effects on extinction of conditioned fear, freezing was measured during tone presentations before and after VNS treatment. Conditioned fear was first measured 1 day after conditioning. After a habituation period of 10 s four tones were presented with an ITI of 3, 4, or 5 min (4 min average), but no footshock was administered during this test session (Day 3; Figure 2A). The session was video recorded and the rats’ behavior was assessed by two independent observers who were blind to treatment conditions. The rats’ freezing response was used as a measure of the CFR and expressed as the percent time spent freezing of total duration of exposure to the conditioned cue. Freezing was defined as a period of complete immobility, characterized by a lowered head, spread paws, and rapid respiration. In order to test whether VNS also has effects on behavior outside of the conditioned response to the tone we also analyzed freezing behavior during the intervals between tone presentations. Analysis of freezing behavior during the ITIs was done in the same manner as that for the CFR to the CS described above.

### Extinction training and VNS/Sham treatment

Extinction trials were administered in the same context as training and testing trials. On Day 4, rats were presented four tones in the absence of footshock. During this treatment trial, VNS (30 s duration, 0.4 mA, 500 μs pulse width at 30 Hz, starting 150 ms before the onset of the tone) or sham-stimulation overlapped with the conditioned 30 s tone. These VNS parameters were selected because they were optimized for enhancing memory consolidation in rats (Clark et al., 1998). Another group received extended extinction (EE) training but no VNS. Rats in this group received 5× the number of tone presentations (i.e., 20 tones) during the treatment trial (Day 4, Figure 2A). Finally, to control for unspecific effects of VNS on synaptic plasticity, one group of animals that was not fear conditioned received the same amount of VNS in their home cages as the experimental group during extinction training.

### Posttreatment conditioned fear test

On Day 5, freezing was assessed in all fear-conditioned animals as described above for the initial conditioned fear test; VNS and sham-stimulation were not administered and the level of freezing to the tone, in the absence of footshock, was again measured. Post-treatment freezing was analyzed as percent of each individual rat’s CFR.

### *In-vivo* electrophysiological recordings

On day 6, evoked field potentials (EFPs) were recorded in the BLA (D/V: 7.2, A/P: 2.7, M/L: 4.9 from bregma) of isoflurane-anesthetized rats using glass microelectrodes (2M KCl; 1–2 MOhms resistance). A bipolar matrix stimulation electrode (FHC, Bowdoin, ME) was placed in the IL region of the vmPFC (D/V: 4.6, A/P: 3.0, M/L: 0.7 from bregma). The width of the stimulation pulse was set to 0.3 ms and the stimulation intensity corresponded to 40% of the minimum current intensity that evoked a maximum field response (based on an input–output curve determined before collection of baseline data). Evoked responses were amplified using a Model 1600 Neuroprobe Amplifier (A-M Systems) and a BMA 200 Portable Bioamplifier (CWE, cwe-inc.com). Signals were digitized using a CED 1401 interface (Cambridge Electronic Design, Cambridge, England) and analyzed using Spike-2 (CED) and Axograph-X (Axograph Scientific, New South Wales, Australia). Data were collected every 15 s and averaged every 1 min for analysis. Baseline data were collected for a minimum of 10 min before synaptic plasticity was induced. The high-frequency stimulation (HFS) protocol used three bursts of 100 pulses at 50 Hz (2 s), with 20 s inter-burst intervals at the minimum current intensity that evoked the maximum field response. The low-frequency stimulation (LFS) protocol consisted of 900 pulses at 1 Hz. The amplitude of the EFP was measured as the difference between the mean of a 5 ms window before the stimulation artifact and the mean of a 5 ms window around 20–25 ms after the stimulation artifact, corresponding to the negative peak of the field potential. Data were normalized to baseline and the average of a 10 min baseline was set as 100% and the 10 min period 40–50 min after plasticity induction was used to analyze long-term plastic changes.

### Histology

After the electrophysiological recordings, the stimulation and recording sites were marked by passing anodal currents (10 mA for 3 s and 10 mA for 2 min) through the electrodes. Rats were overdosed with urethane (3 g/kg) and then transcardially perfused with 0.9% saline followed by 4% paraformaldehyde in PBS. Coronal sections were cut on a freezing microtome and Nissl-stained for the identification of stimulation and recording sites.

### Data analysis

Single trial behavioral data were analyzed with an ANOVA and pair-wise treatment group comparisons used Tukey-Kramer *post hoc* tests. Multiple trial experiments were analyzed with a partially repeated ANOVA (treatment group × trial), and group differences were determined using Tukey-Kramer *post hoc* tests. Changes in post-stimulation EFP amplitudes were compared using partially repeated-measures ANOVA with a treatment group × time interaction.

## Results

### Vagus nerve stimulation enhances extinction memory

Animals in the three groups given extinction treatment (Figure 2B; VNS, *n* = 14; sham, *n* = 16; and EE, *n* = 10) showed a similar degree of CFR on the first test day (Day 3). An ANOVA revealed no significant group differences in CFR on the day before treatment (*F*_(2,37)_ = 1.332, *p* = 0.276). Analysis of the CFR measured 1 day after treatment (Day 5) revealed a significant main effect across treatment groups (*F*_(2,37)_ = 20.682, *p* < 0.001). Consistent with previous findings, the CFR of rats given only four non-reinforced exposures to the conditioned tone without VNS treatment on Day 4 changed only marginally, indicating no reduction in conditioned fear after the treatment trial. In contrast, a significant reduction in the percentage of CFR was seen in the group of rats in which a single extinction session (4 tone pairings) was paired with VNS (Tukey-Kramer, *p* < 0.001 vs. sham), as well as in the group of animals that received EE training (EE, 20 tone pairings) (Tukey-Kramer, *p* = 0.002 vs. sham). The magnitude of the reduction in CFR did not differ between the VNS and EE groups (Tukey-Kramer *p* = 0.105; Figure 2B). Similarly, an ANOVA showed no significant differences in freezing during the ITI on Day 3 (*F*_(2, 37)_ = 2.858, *p* = 0.070), but after treatment a significant main effect was found across treatment groups (*F*_(2, 37)_ = 17.694, *p* < 0.001) on Day 5. Rats treated with VNS showed reduced freezing behavior when compared to both sham and EE groups (Tukey-Kramer, *p* < 0.05 vs. sham and EE); in contrast, rats in the EE group did not differ in freezing behavior from sham stimulated animals during Day 5 ITIs (Tukey-Kramer, *p* = 0.308; Figure 2C). Taken together, these findings indicate that VNS facilitated extinction of conditioned fear, bringing it to the level achieved by EE training, consistent with our previous results (Peña et al., 2013), and in addition, VNS also facilitated extinction to the context as evidenced by reduced freezing behavior during the inter-tone intervals.

### Vagus nerve stimulation modulates plasticity in the IL-amygdala pathway

**Figure 3:**
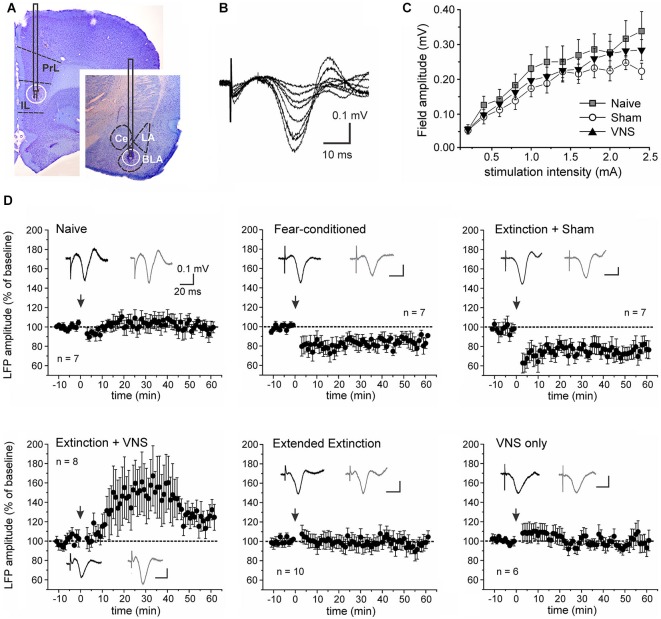
**Vagus nerve stimulation alters metaplasticity in the IL-BLA pathway**. **(A)** Representative stimulation and recording sites in the IL and BLA. **(B)** Single-pulse stimulation targeted to the infralimbic (IL) PFC elicited negative field potentials in the BLA that peaked after 20–25 ms. Representative traces (average of 10 consecutive sweeps) of an input-output curve from a sham-stimulated rat. **(C)** Input-output curves from naïve rats (*n* = 7), fear-conditioned sham-stimulated rats (*n* = 7) and rats receiving VNS during extinction (*n* = 8). **(D)** Synaptic plasticity in the IL-BLA pathway in response to short burst stimulation in six groups of rats. Top row: In naïve rats burst stimulation does not induce synaptic plasticity. In fear-conditioned only and rats that received sham-stimulation during a single extinction session (4 tone presentations), burst stimulation induces LTD. Bottom row: In rats that received extended extinction training (20 tone presentations) and showed reduced freezing behavior, LTD induction is abolished. In rats treated with VNS during a single extinction session (4 tone presentations), synaptic strength is further potentiated, leading to LTP. This effect on synaptic plasticity appears to be context-specific as VNS stimulation by itself (VNS only) in untrained animals receiving VNS in their home cages did not affect plasticity in the IL-BLA pathway. PL, prelimbic cortex; BLA, basolateral nucleus of the amygdala, LA, lateral nucleus of the amygdala; CE, central nucleus of the amygdala.

Stimulation of the IL elicited negative field potentials in the BLA, which peaked after 20–25 ms (Figure 3). The current-voltage relationship between stimulation intensity in the IL and EFP amplitude in the BLA did not significantly differ between fear-conditioned sham-stimulated rats and rats that received VNS during extinction training (*F*_(2,30)_ = 0.435, *p* = 0.651; Figure 3C). To further examine learning- and VNS-induced synaptic plasticity in the IL-BLA pathway we used short burst stimulation (HFS) of the IL. A repeated measures ANOVA showed a main effect of treatment (*F*_(5,30)_ = 5.983, *p* = 0.0003). Consistent with previous reports (Maroun, 2006), this protocol did not induce synaptic plasticity in naïve rats (*n* = 8, +3.08 +/− 3.17% change, *F*_(1,12)_ = 0.106, *p* = 0.75). In rats given 2 days of fear conditioning without extinction training (FC, *n* = 7) and in rats that received only sham-stimulation during the treatment phase of extinction (*n* = 10), resulting in no reduction of the freezing response, HFS of the IL induced LTD of the EFP (FC −15.35 +/− 2.04% change, *F*_(1,12)_ = 7.855, *p* = 0.016; sham −25.28 +/− 2.39% change, *F*_(1,18)_ = 17.6, *p* = 0.001). In contrast, HFS induced LTP (+30.44 +/− 5.41% change, *F*_(1,14)_ = 6.6063, *p* = 0.022) in the IL-BLA pathway of rats given a single extinction session paired with VNS (*n* = 8) (Figure 3D). To determine whether the change in the sign of plasticity in the IL-BLA pathway was due to extinction of conditioned fear, the pairing of extinction training with VNS, or VNS alone, we tested two control groups. Increasing the amount of tone exposures during extinction (EE group) reversed the LTD in the IL-BLA pathway seen in fear conditioned and sham-stimulated rats (−0.64 +/− 2.62% change, *F*_(1,18)_ = 0.008, *p* = 0.931). On the other hand, applying VNS outside of the fear-conditioning context (i.e., applying VNS to untrained, never fear-conditioned rats in their home cages; *n* = 6) did not alter the ability to induce plasticity in the IL-BLA pathway (−4.30 +/−1.94% change, *F*_(1,10)_ = 0.633, *p* = 0.455; Figure 3D).

**Figure 4:**
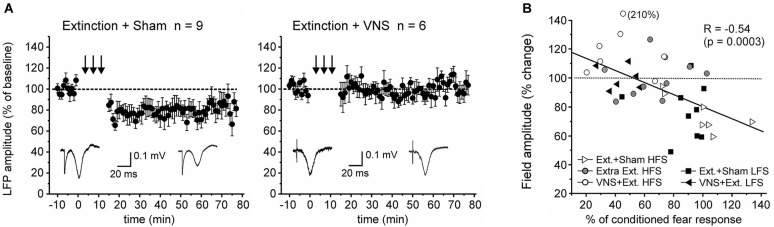
**Pairing VNS with extinction prevents the induction of LTD. (A)** In sham-stimulated rats (*n* = 9) low-frequency stimulation resulted in LTD in the IL-BLA pathway. In rats in which extinction training was paired with VNS (*n* = 6), induction of LTD was inhibited. **(B)** Plot of the correlation between the amount of conditioned fear response on day 6 and the change in EFP amplitude following high-frequency (HFS, c.f. Figure 2) or low-frequency stimulation (LFS).

Finally, we tested whether context-specific modulation of synaptic plasticity is frequency-dependent. To this end we used a low frequency stimulation protocol that has previously been shown to induce robust LTD in the IL-BLA pathway in naïve rats (Maroun, 2006). A partially repeated-measures ANOVA revealed a significant difference in the EFP following LFS between rats which received VNS during extinction (*n* = 6) and sham-treated rats (*n* = 9) (*F*_(1,10)_ = 5.156, *p* = 0.041). In sham-stimulated rats LFS induced LTD (−21.7 +/− 2.6% change, *F*_(1,16)_ = 11.662, *p* = 0.004), but this was suppressed in rats that received VNS during extinction (+1.9 +/− 2.4% change, *F*_(1,10)_ = 0.122, *p* = 0.734), indicating that under these conditions VNS similarly reversed fear-conditioning-associated plasticity in the IL-BLA pathway (Figure 4).

## Discussion

Consistent with our previous results (Peña et al., 2013), we found that administration of VNS during a single session of exposure to conditioned cues facilitates extinction of conditioned fear. Here we expand on these behavioral findings by examining the effects of fear conditioning and extinction on synaptic plasticity in the IL-BLA pathway. Both the IL and the BLA are implicated in fear conditioning and extinction (Marek et al., 2013). Here, we found evidence that fear conditioning and extinction produce metaplasticity in the IL-BLA pathway, modulating conditions for the induction of synaptic plasticity. Fear conditioning predisposed synapses toward depression and extinction reversed this effect. Importantly, VNS delivered during extinction training further potentiated the evoked responses in the BLA, resulting in LTP in the IL-BLA pathway. Thus, VNS enhances extinction and has lasting effects on synaptic plasticity in a pathway crucial for extinction learning. Neural activity in the vmPFC is increased during recall of extinction memory (Milad and Quirk, 2002), and HFS of the IL following retrieval of a conditioned fear memory enhances subsequent fear extinction learning (Maroun et al., 2012). On the other hand, previous reports have shown that successful fear extinction reduces the efficacy of excitatory synaptic inputs from the vmPFC to the BLA (Vouimba and Maroun, 2011; Cho et al., 2013). The reasons for this discrepancy are not clear, but may reflect compensatory mechanisms and cell type specific projections of the vmPFC to the BLA and the intercalated cells, respectively (Cho et al., 2013). Here, using recordings in anesthetized rats more than 24 h after the last behavioral testing, we observed no significant differences in the input-output relationship of EFPs before plasticity-induction between animals that received sham-stimulation or VNS. Nevertheless, behavioral training altered the conditions for the induction of synaptic plasticity in this pathway. Consistent with a previous report (Maroun, 2006) we found that the pathway from the IL to the BLA is resistant to the induction of LTP in naïve animals. However, following fear conditioning, burst stimulation of the IL induced LTD in the BLA of fear conditioned and sham-stimulated rats. Extended extinction training or VNS during a single extinction session reversed this plasticity. In VNS-treated rats this resulted in the promotion of LTP in response to HFS. In a follow-up experiment we tested whether pairing VNS with extinction generally promoted LTP within the circuit or, alternatively, can also enhance LTD induced by low frequency stimulation of the IL. Under these conditions LTD induction was inhibited, suggesting that pairing VNS with extinction facilitates the ability of IL stimulation to potentiate synapses in the BLA. Importantly, this shift occurred only in combination with behavioral training. VNS provides network-specific modulation of experience-driven synaptic actions to promote lasting circuit-specific plasticity (Engineer et al., 2011; Porter et al., 2012). Accordingly, and consistent with our previous behavioral data (Peña et al., 2013), VNS only altered the plasticity in the pathway between the IL and the BLA when it was delivered in an extinction context. In contrast, VNS delivered to untrained animals in the home cage had no effect. It is interesting to note that VNS paired with extinction training, unlike EE training by itself, also facilitated the extinction of freezing behavior outside of the presentation of the CS. Because animals underwent extinction training in the same context as the auditory fear conditioning, this change of behavior during the ITI might be an indicator that VNS facilitated generalization of extinction learning to the context. It is tempting to speculate whether these behavioral differences are also reflected in the different magnitudes of potentiation of the EFP seen after (extended) extinction training by itself and extinction training paired with VNS, respectively.

The mechanisms through which VNS modulates activity in the central nervous system are poorly understood, but proposed mechanisms include alteration of norepinephrine (NE) release by projections from the nucleus tractus solitaris to the locus coeruleus (LC), elevated levels of inhibitory GABA related to vagal stimulation, and inhibition of aberrant cortical activity by reticular system activation (Ghanem and Early, 2006; Manta et al., 2009). Thus VNS may modulate cortical plasticity and memory via the synergistic action of multiple neuromodulators, which also include acetylcholine, serotonin, and brain-derived neurotrophic factor (Dorr and Debonnel, 2006; Nichols et al., 2011; Manta et al., 2013). Acute VNS increases NE and serotonin release in both the medial PFC and the amygdala (Hassert et al., 2004; Roosevelt et al., 2006; Manta et al., 2009) and enhances synaptic transmission in the hippocampus (Zuo et al., 2007; Shen et al., 2012; Ura et al., 2013). Norepinephrine has previously been shown to be involved in the modulation of fear expression. Locus coeruleus neurons fire in response to unexpected changes in stimulus-reinforcement contingencies (Sara and Segal, 1991; Sara, 2009). Lesions of the NE projections from the LC to the forebrain impair the extinction of active avoidance without altering acquisition or retention of the original learning (Fibiger and Mason, 1978; Mason and Fibiger, 1978). Whereas consolidation of conditioned fear depends on β-adrenoceptor activation within the BLA (McGaugh, 2002), previous evidence suggests a role for both α- and β-adrenergic receptors in the medial PFC in memory consolidation of extinction training (LaLumiere et al., 2010; Mueller and Cahill, 2010; Smith and Aston-Jones, 2011; Buffalari et al., 2012). Thus pairing VNS with extinction may set the stage for synaptic plasticity and consolidation of extinction and through β-adrenergic receptor-PKA mediated phosphorylation of AMPA receptors, marking them for membrane insertion (Mueller and Cahill, 2010; Shen et al., 2012). Similarly, the relative activation of neuromodulator receptors coupled to adenylyl cyclase and phospholipase C may result in phosphorylation of postsynaptic glutamate receptors at sites that specify induction of LTP or LTD, respectively. Thus, cholinergic and adrenergic neuromodulation associated with the behavioral state of the animal can control the gating and the polarity of cortical plasticity (Seol et al., 2007).

Several anxiety disorders, including PTSD, are associated with poor vagal tone and an altered balance of activity between the vmPFC and amygdala (Friedman, 2007; Milad and Quirk, 2012). Activation of the vmPFC during therapy is correlated with positive patient outcomes (Bryant et al., 2008). A hallmark of anxiety disorders is impaired extinction of traumatic memories (Jovanovic et al., 2010; Norrholm et al., 2011). Our findings that VNS enhances the extinction of a conditioned fear and changes synaptic strength in the IL-BLA pathway suggest that VNS can be used to overcome an insufficient vagal response to conditioned cues in order to enable the consolidation of extinction memory. Thus VNS, which is clinically approved for the treatment of depression and the prevention of seizures, could be used as an adjunct treatment to exposure therapy because it produces pairing-specific plasticity and enhances the effect of exposure on extinction of conditioned fear responding.

## Financial disclosures

The authors received research and salary support from Microtransponder Inc. to collect preliminary data for this project.

## Conflict of interest statement

Dr. McIntyre is an author of a patent entitled “Enhancing fear extinction using vagus nerve stimulation”.
